# Human herpes virus 8-unrelated primary effusion lymphoma-like lymphoma diagnosed by fluorodeoxyglucose positron emission tomography/computer tomography and laparoscopy

**DOI:** 10.3892/ol.2013.1731

**Published:** 2013-12-05

**Authors:** WENZHI WU, JINGYUN LIU, WANDONG HONG

**Affiliations:** 1 Department of Gastroenterology and Hepatology, The First Affiliated Hospital of Wenzhou Medical University, Wenzhou, Zhejiang 325000, P.R. China; 2 Department of Ultrasound, The First Affiliated Hospital of Wenzhou Medical University, Wenzhou, Zhejiang 325000, P.R. China

**Keywords:** primary effusion lymphoma, ascites, positron emission tomography, computer tomography

## Abstract

Human herpes virus 8-unrelated primary effusion lymphoma (PEL)-like lymphoma is a rare type of large B cell lymphoma. This report presents the case of a male with abdominal pain and distension who was found to have massive ascites and enhanced peritoneum, mesenterium and greater omentum on enhanced computer tomography (CT) scan with negative ascitic cytology. The diagnosis of PEL-like lymphoma was established by fluorodeoxyglucose (FDG) positron emission tomography (PET)/CT and laparoscopic biopsy of the greater omentum. To the best of our knowledge, this is only the second case report to describe FDG PET/CT presentations of PEL-like lymphoma, and the first case report to use laparoscopy for diagnosis.

## Introduction

Primary effusion lymphoma (PEL) is a rare type of large B-cell lymphoma characterized by lymphomatous effusion of body cavities without lymphadenopathy or organomegaly. PEL often occurs in patients with human immunodeficiency virus (HIV) and/or human herpes virus type 8 (HHV-8) infections (1). However, patients have been reported with HHV-8-negative and HIV-negative PEL with high expression of B cell markers. This is described as HHV-8-unrelated PEL-like lymphoma (2). To date, only one case report has described the presentation of PEL using fluorodeoxyglucose (FDG) positron emission tomography (PET)/computer tomography CT) (3), and there has been no such study for PEL-like lymphoma. To the best of our best knowledge, all cases of PEL or PEL-like lymphoma reported in the literature were diagnosed by ascitic cytology. This report presents a case of PEL-like lymphoma with negative ascitic cytology, which was identified by FDG PET/CT and ultimately confirmed by laparoscopic biopsy of the greater omentum. Written informed consent was obtained from the patient’s family.

## Case report

A 39-year-old male was referred to the First Affiliated Hospital of Wenzhou Medical College (Wenzhou, China) with a one-week history of abdominal pain and distension. Laboratory tests revealed a white blood cell (WBC) count of 10.1×10^9^ cells/l and hemoglobin levels of 12.8 g/dl. Aspartate aminotransferase levels were 14 U/l and alanine aminotransferase levels were 70 U/l. Tests for hepatitis markers, Epstein-Barr virus (EBV), HHV-8, HIV and tumor markers were negative.

The WBC count of the ascitic fluid was 640,000 cells/ml: 5% polymorphonuclear leukocytes, 71% lymphocytes and 24% abdominal cells. Bacterial cultures were negative. The ascitic effusion test for HHV-8 using polymerase chain reaction was also negative. Ascitic cytology was performed three times but no malignant cells were found. Gastroscopy and colonoscopy were also normal.

**Figure 1:**
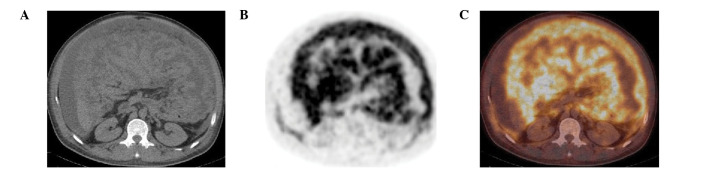
Transaxial view of (A) CT (B) PET scans and (C) fused PET/CT images showing intense FDG uptake in the peritoneum, mesenterium and greater omentum (maximal standardized uptake value, 7). CT, computer tomography; PET, positron emission tomography; FDG, fluorodeoxyglucose.

Enhanced abdominal CT scan showed massive ascites and enhanced peritoneum, mesenterium and greater omentum but no detectable mass or lymphadenopathy. Therefore, the patient underwent FDG PET/CT examination. FDG PET/CT showed FDG uptake in the peritoneum, mesenterium and greater omentum (Fig. 1). No mass or lymphoma cells were detected by whole-body CT, FDG PET or bone marrow biopsy.

**Figure 2:**
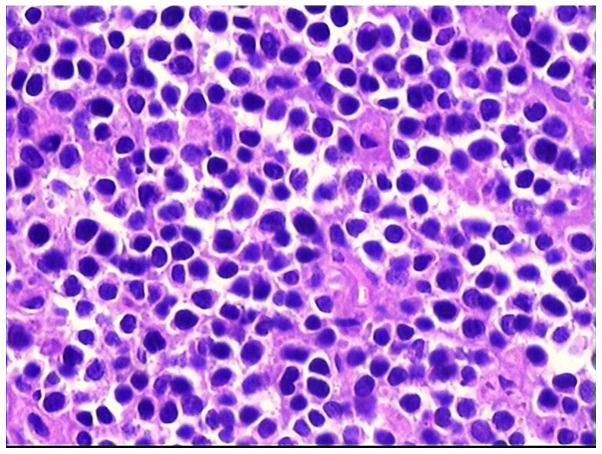
Laparoscopic biopsy specimen containing lymphoma cells exhibiting a B-cell phenotype with large nuclei and abundant cytoplasm (magnification, ×400).

A laparoscopic biopsy of the greater omentum was performed, revealing lymphoma cells with large nuclei and abundant cytoplasm which exhibited a B-cell phenotype (Fig. 2). Immunohistochemical staining revealed that the large atypical cells were positive for cluster of differentiation (CD) 10 (+), CD20 (++++), CD79a (++++), Ki67 (95%+), multiple myeloma oncogene 1 (++) and paired box 5 (++), but negative for anaplastic lymphoma kinase, B cell lymphoma (Bcl) 2, Bcl-6, CD2, CD117, CD21, CD3, CD30, CD34, CD43, CD5, CD56, CD68, CD7, CD99, CD1A, creatine kinase, cyclin-D1, Epstein Barr virus-encoded RNA, epithelial membrane antigen, granzyme B, myeloperoxidase, perforin and terminal deoxynucleotidyl transferase.

The patient was diagnosed with HHV-8-unrelated HIV-negative PEL-like lymphoma (indeterminate phenotype). The patient and his relatives refused chemotherapy and the patient succumbed to PEL-like lymphoma one month later.

## Discussion

PEL is often associated with HHV-8 and occurs most frequently in immunodeficient states (1). However, the etiology of HHV-8-unrelated PEL-like lymphoma is unknown. Hepatitis C virus (HCV) infection has been suggested to induce persistent antigenic stimulation that results in B-cell clonal expansion (4). The reported rate of association of PEL-like lymphoma with HCV is 30–40% (5). However, in the majority of patients with HHV-8-unrelated PEL-like lymphoma, as was the case in the present study, no known pathogens, including HIV, EBV, HCV, or iatrogenic immunodeficiency, can be identified (4).

The diagnosis of PEL-like lymphoma is primarily based on cytological evaluation of fluid material by immunohistochemistry or flow cytometry. However, no malignant cells were found despite the fact that ascitic cytology had been performed three times in the current case study. Therefore, this patient underwent FDG PET/CT examination and laparoscopic omentum biopsy. As a useful non-invasive diagnostic tool, FDG PET supplements conventional imaging in diagnosis of peritoneal disease, as this technique can detect lesions not identified by CT (6). Makis *et al*(3) first described the appearance of F-18 FDG PET/CT in a patient with hepatitis C-related PEL. Results showed a marked increased F-18 FDG uptake in the pleura and peritoneum on the left side. The current case report demonstrates increased F-18 FDG uptake in the peritoneum, mesenterium and greater omentum. As FDG is taken up by macrophages, granulation tissues and inflammatory tissues, in addition to tumor cells, intense F-18 FDG uptake in the peritoneum may also occur in tuberculous peritonitis, peritoneal carcinomatosis, and peritoneal mesothelioma (3). To date, no reliable PET/CT criteria have been established for differential diagnosis of these diseases (6).

There is no consensus on the optimal treatment of HHV-8-unrelated PEL-like lymphoma due to the small number of published reports. Cyclophosphamide hydroxydaunorubicin oncovin prednisone-like regimen (1) or rituximab-containing regimen (2) have frequently been administered in these cases. Although the prognosis of HIV-negative HHV-8-unrelated PEL-like lymphoma patients is better than the HIV-positive PEL group (1), in this case, the prognosis was still poor and the patient succumbed to PEL-like lymphoma one month following diagnosis.

In conclusion, PET/CT and laparoscopic biopsy may be useful diagnostic tools for PEL-like lymphoma when the origins of ascites cannot be determined by general ascitic examination or conventional imaging tests, such as CT scans.
